# Cognitive inhibition abilities explain inter-individual variability in gender-space associations

**DOI:** 10.3389/fpsyg.2023.1130105

**Published:** 2023-05-17

**Authors:** Aitor Calvente, Carmen Noguera, Dolores Álvarez, Sergio Fernández, Isabel Carmona

**Affiliations:** ^1^Department of Psychology, University of Almería, Almería, Spain; ^2^CEINSA, Health Research Center, University of Almería, Almería, Spain

**Keywords:** mental representation of gender, spatial bias of gender, individual differences, cognitive inhibition, conscious/non-conscious perception

## Abstract

There is a great deal of research describing the close association that exists between numerical and spatial representations, illustrating the SNARC (Spatial-Numerical Association of Response Code) effect. This effect signals the spatial mental representation of small numbers to the left and larger numbers to the right, coinciding with the direction of reading and writing. Subsequent research has found a similar spatial representation for other stimuli (e.g., size of objects and animals, and words associated with time). Some of these spatially represented stimuli are social in nature, even suggesting a spatial mental organization of stimuli based on gender (e.g., the upper part of a vertical axis for males and the lower part for females). The aim of the present study was threefold (1) to replicate and extend results on the existence of a mental gender line (as a function of response hand: female-left hand and male-right hand) when responding simply to gender of stimuli; (2) to explore the influence of inhibitory control; and, (3) to determine whether gender-space associations depend on the explicit or implicit nature of a gender task. Three experiments were designed to pursue these objectives. In Experiment 1, female, male and neutral faces and names were displayed, and the participants were asked to identify their gender. Experiment 2, which also included a Stroop task, followed the same procedure as Experiment 1, but displayed objects that could be designated as female or male and others not related to any gender. Finally, in Experiment 3, in which participants were asked to respond to the direction of an arrow, object gender was not relevant to the task. Consistent with previous research and confirming our hypotheses, the results showed a spatial mental representation of the stimuli based on gender in all three experiments, regardless of whether the stimulus was consciously perceived. Moreover, inhibitory ability showed a relationship with the gender-space line effect. The contributions and implications of this study are discussed, as are possible limitations and future lines of research.

## Introduction

The close relationship between certain abstract or concrete concepts, such as time, valence or social power, number or size, or spatial representations has been widely explored in recent decades (Schubert, 2005; Pitt and Casasanto, 2020). This research has shown a motor prime response depending on the mental representation of the concepts. For instance, positive valence is associated with rightward space, and negative valence with leftward space in right-handers, while left-handers show the opposite pattern (Casasanto, 2009; Kong, 2013). However, the mental representation of other concepts, such as number, size or time are not influenced by handedness, but rather the direction of reading and writing. Santiago et al. (2007) asked western participants to classify words presented to the left or right of the central fixation point, according to whether they referred to the past (e.g., yesterday) or the future (e.g., tomorrow). Participants responded faster with the left hand for words with a past meaning and with the right hand for words with a future meaning, regardless of spatial location, suggesting a spatial representation of time. Fuhrman and Boroditsky (2010) compared the performance of English and Hebrew-speaking participants using a task consisting of ordering pictures representing different phases of a temporal sequence. The English-speaking participants, who read from left to right, ordered the images according to reading direction, while Hebrew speakers did it in a right-to-left direction. Other aspects, such as size, also seem to be represented spatially, as shown in the study by Sellaro et al. (2015), in which smaller objects and animals were placed on the left and larger ones on the right. What is more, even though participants were not explicitly instructed to process the stimuli by size, this perceptual feature influenced how they responded. This pattern was observed even when the task consisted of classifying stimuli as “living” or “non-living”.

Other studies also provide information on the possible influence of these spatial mental representations, biased by the direction of reading and writing, on social aspects. Presaghi and Rullo (2018) found that participants, influenced by the feeling of group belonging, responded faster with the left hand to the image of a person from the same social group (ingroup), and with the right hand to the image of a person from a different social group (outgroup). This effect was called Spatial Organization of Social Categories (SOSC). Moreover, Maass et al. (2009) (see also Suitner and Maass, 2016) found that participants with strong sexist stereotypes tended to draw men to the left of women in an action scene (e.g., a volleyball match). The authors argue that this response pattern is due to a spatial bias in the representation of social groups consistent with writing direction. In another experiment, they observed that Italian-speaking participants showed a tendency to place agentic groups (men and young people) to the left of less agentic groups (women and older people), whereas Arabic-speakers tended to represent agentic groups to the right. In the context of their theoretical model, Spatial Agency Bias (SAB), this biased representation would be the result of the joint function of two interrelated asymmetries, one from writing direction and the other from subject-object order. Other works reported a spatial representation of information related to gender on a vertical axis. For instance, Zhang et al. (2014) and Zarzeczna et al. (2020) observed that participants placed the male gender at the upper end, and the female gender at the lower end.

An effect that has received much attention is the serial representation of numbers over a mental line from left to right, which is known as the SNARC effect (Spatial-Numerical Association of Response Codes). One of the pioneering works in this field was Dehaene et al. (1993), which, through a series of experiments in which participants were asked to classify numbers as odd or even, observed an interaction between the response hand and the magnitude of the number. Participants showed shorter latency time to respond with the left hand to small numbers, and with the right hand to large ones, than when the response-hand/numerical-magnitude association was left/large, right/small. Subsequent studies showed that the direction of this mental number line representation depended on cultural factors, such as the direction of mother tongue reading and writing (Shaki et al., 2009). In addition to cultural factors, several authors deem it essential to consider other aspects to explain this effect, such as the nature of the numerical task, age or interference control (Wood et al., 2008; Hoffmann et al., 2014; Wu et al., 2020).

Hoffmann et al. (2014), for instance, examined the roles played by age, processing speed, working memory, and cognitive inhibition (as a source of inter-individual variability) to demonstrate the SNARC effect. For this purpose, participants performed several tasks which included: a speed task to assess general processing speed, a pencil and paper version of the Stroop test, a computerized version of the Simon task to measure cognitive interference and inhibition, a digit span task to examine short-term and working memory, and an odd-even number sorting task to measure the SNARC effect. The results showed a relationship between response times to sort numbers and Stroop interference, age, and processing speed. The magnitude of the SNARC effect, in terms of difference between the response time with the left hand to a number and the response time with the right hand to the same number, was greater in older participants among both those with slower processing speed and those who showed lower inhibition ability in the Stroop task. In contrast, no relationship was observed between the occurrence of the effect and Simon-type interference, working memory and short-term memory.

Similarly, Georges et al. (2018) confirmed the role of inhibition ability in the inter-individual variability observed in the SNARC effect, and, furthermore, extended these results by considering that this relationship depends on explicit or implicit processing of number magnitude. The authors included the same Stroop-like task and the General Processing Task described in Hoffmann et al. (2014), in addition to a flanker task (Eriksen and Eriksen, 1974) to measure the control of distracting information interference. Two versions of the number task were employed: a task in which participants were instructed to classify numbers as “> or <5”, and another in which they were asked to classify them as “odd or even”. In the first case, the perception of the magnitude of the number would be intentional or explicit, while in the second case an implicit or unintentional perception of magnitude would occur because magnitude is an irrelevant dimension for that task. The results showed an interesting pattern: participants with weaker interference control in the Stroop task presented greater SNARC effect, but only when the odd-even number task was employed. In contrast, when magnitude becomes the relevant dimension, as it is in the numerical task, a stronger SNARC effect is associated with better interference control in the flanker task.

The authors theorize that these results are due to the nature of the interference involved in the paradigms for assessing cognitive inhibition. These paradigms are verbal in the Stroop task and spatial in the Flanker task, coinciding with the cognitive processes responsible for the appearance of the SNARC effect in each numerical task, which are verbal in nature when implicit and spatial in nature when explicit or intentional. Thus, in the flanker task, which involves responding to a target “flanked” by irrelevant stimuli, a greater ability to inhibit the irrelevant stimuli could also mean a better ability to inhibit the numbers (and their magnitudes) flanking the target number represented on the mental number line. However, when the criterion for sorting numbers is different from their magnitude (e.g., an odd-even task), the observation of the SNARC effect could be due to a worse (or less effective) inhibition of the activated magnitude-related information.

The studies by Hoffmann et al. (2014), and Georges et al. (2018) bring to light the importance of inter-individual variability in the strength of number-space associations, highlighting individual differences in the ability to resist interference from irrelevant information, and the explicit or implicit nature of the numerical task. Indeed, it would be interesting to know if these effects can extend to other types of spatial representations like the gender-space association cited above (Maass et al., 2009; Suitner and Maass, 2016; Presaghi and Rullo, 2018). To our knowledge, it remains unclear whether this type of spatial organization also includes objects typically considered to be masculine or feminine, and whether the gender-space association effect can be modulated by the inhibitory abilities of participants.

### Current study

The aim of the present study was threefold: (i) to replicate and extend results on the existence of a mental gender line; (ii) to explore the influence of inhibitory control, assessed by using the classic Stroop task, on gender-space association; and (iii) to determine whether gender-space associations depend on the explicit or implicit nature of a gender task. To achieve these goals, three novel experiments were designed. In Experiment 1, faces and names were displayed in the center of the screen, and participants were asked to identify the gender (masculine vs. feminine) of each item (explicit gender task). Experiment 2 included an explicit object classification task and a Stroop task to extend the possible occurrence of a gender-space association when classifying objects with gender implication (e.g., a lipstick) and to examine the relationship between the ability to inhibit distracting information and the strength of the gender-space association. Experiment 3 was conducted to explore whether the gender spatial representation effect could be observed when gender perception was not relevant to the task, and whether individual differences in inhibitory capacity influence the strength of occurrence of the gender spatial effect, as suggested by Hoffmann et al. (2014), and Georges et al. (2018). This experiment included two tasks, the same Stroop task employed in Experiment 2, and the Arrow task. In the latter, gender object images (e.g., lipstick) were followed by arrows pointing to the left or right. Participants were asked to respond to the direction of the arrow. All stimuli were presented under two perceptual processing conditions, conscious (delayed masking condition) and non-conscious (immediate masking condition). The masking condition was manipulated to explore whether the gender-space mental representation could be activated in both conditions of awareness.

To the extent that writing direction influences our social cognition (Maass et al., 2009; Suitner and Maass, 2016), an interaction between the response hand and stimulus gender was expected. In contrast to previous studies on social cognition-space associations, in the gender task, the stimuli were presented individually in the center of the screen, in the absence of context or an action scene, so the response pattern could be partially biased by the direction of writing, but not by the subject-object order, as established by the SAB model (Maass et al., 2009; Suitner and Maass, 2016). Furthermore, the stimuli were presented in a novel way, that is, under conditions of conscious and non-conscious perception, which made it possible to explore whether the spatial representation of gender could be observed even when information was processed without awareness.

The results obtained in the context of the SNARC effect establish a relationship between the capacity to inhibit distracting information and the strength of the number-space association. This relationship seems to depend on the nature (explicit vs. implicit) of the numeric task, and on the type of paradigm used to assess inhibition (Hoffmann et al., 2014; Georges et al., 2018). Taking this into account and employing a Stroop-like task to measure individual differences in inhibition, we expected to find a larger gender-space effect for those participants with lower inhibition ability, in both conscious and non-conscious perceptual conditions. In other words, the presentation of a gender-stereotyped object would activate the gender-space representation and, thus, the congruent response hand schema. However, this activated schema could conflict with the (hand of) response to the target arrows. Those individuals with less ability (or efficiency) to inhibit the gender-activated schema would take longer to settle said conflict and, therefore, would show a greater difference of response latency between congruent and incongruent gender-space/arrow-direction trials.

## Materials and methods

### Participants

Participants were recruited from the Psychology Degree program, receiving credit toward course requirements as compensation for their collaboration. All participants signed a written consent form after receiving an explanation about the nature of the research, but without disclosing the hypotheses. All of them had normal or correct to normal vision. The study was approved by the Ethical Committee of the University of Almería and conducted in accordance with the Helsinki Declaration.

Experiment 1 involved 44 undergraduates (22 women) with a mean age of 24.6 years (*SD* = 8.86), 36 right-handers, 6 left-handers and 2 ambidextrous individuals. In Experiment 2 the sample was comprised of 30 college students (sixteen women; *M* = 22.63, *SD* = 4.03), 31 right-handers, 6 left-handers and 1 ambidextrous individual. Another sample of 20 volunteers was selected to participate in Experiment 3 (nine men; *M* = 21.81, *SD* = 2.34), consisting of 19 right-handers and 1 left-hander.

Sensitivity power analyses, given the alpha and power values, were performed with G^*^Power software, version 3.1.9.2 (Faul et al., 2007) to determine the minimum effect size that could reliably be detected from the sample size in each experiment. In Experiment 1, with an alpha = 0.05, a large effect size (d = 0.81) and total sample size = 44, the analysis revealed statistical power >0.99, and a minimum effect size (d = 0.48). In Experiment 2, with an alpha = 0.05, a medium effect size (d = 0.53) and total sample size = 30, the analysis revealed statistical power >0.99; and a minimum effect size of 0.46. Finally, in Experiment 3, with an alpha = 0.05, a medium effect size (d = 0.60) and total sample size = 20, the analysis showed statistical power >0.90, and a minimum effect size of 0.56. The effect size was performed from the minimum partial eta square of main effect in each experiment.

Regarding the *T*-test, the sensitivity analyses showed the following results: in Experiment 1, a medium effect size of 0.62 was revealed, with statistical power higher than 0.80 and a minimum effect size of 0.57; in Experiment 2, a large effect size of 0.99 was revealed, with statistical power higher than 0.99 and a minimum effect size of 0.74; and in Experiment 3, a medium effect size of 0.56 was exhibited, with statistical power higher than 0.90 and a minimum effect size of 0.53.

All of these results demonstrate that the design of our study has sufficient statistical sensitivity.

At the initial phase of the study participants were asked to perform the gender stereotypes questionnaire (Castillo and Montes, 2007). All participants assigned positive adjectives to the female gender and negative ones to the masculine gender. Also, 82% considered themselves feminists.

### Stimuli and apparatus

A set of 45 faces was selected from The Chicago Face database (Ma et al., 2015), while the set of 45 names were randomly selected from different lists of the most common names (Statistical National Institute database of Spain, INE). Two forms, elaborated by using Google Forms, were administered to 50 participants to rate how masculine or feminine they perceived these faces and names on a 1–10 numeric rating scale (1 = most feminine to 10 = most masculine). Thus, a total of 20 faces and 20 names valued as the most stereotypic items were selected. Those with values between 4 and 6 were identified as neutral. For the object classification task, a set of 60 CC0-licensed images of objects was used. As before, all of them were included in a form to be rated on a scale of 1–7 by a sample of 36 volunteers. The 10 objects perceived as most feminine (e.g., a lipstick), and the 10 as most masculine (e.g., an electric shaver) were used. Another set of 10 objects close to a score of four were classified as neutral (e.g., a clip), since they can be used interchangeably by men and women. None of the participants of this phase (stimuli classification and selection) performed any experimental task. Selected stimuli are included as Supplementary material.

The size of the images was 3 × 3° of visual angle for faces, objects and masks. The length of names ranged from five to seven characters, and they were displayed with 2° of visual angle, in Times New Roman, 18, black font. The length of arrows was 2° of visual angle, and they were displayed 2° above or below the fixation point in black font. All stimuli were displayed on a 19-inch screen with white background. The masks were made by a random combination of colored pixels, using the free d-Code application. The E-prime v3.0 software (Psychology Software Tools, Pittsburgh, PA) was used to design all the tasks, and record the responses.

### Experiments tasks and procedure

#### Experiment 1 (face and name classification by gender tasks)

##### Face classification by gender task

Participants were instructed to classify faces as male or female by pressing the “S” key (located on the left side of the keyboard) with the left hand, or the “L” key (located on the right side) with the right hand. The task was divided into two experimental blocks so that, in one block the “S” key was associated with one gender (e.g., male), and the “L” key with the other (e.g., female). In the other block, the key-gender association was the opposite. The order of presentation of the blocks was counterbalanced.

**Figure 1:**
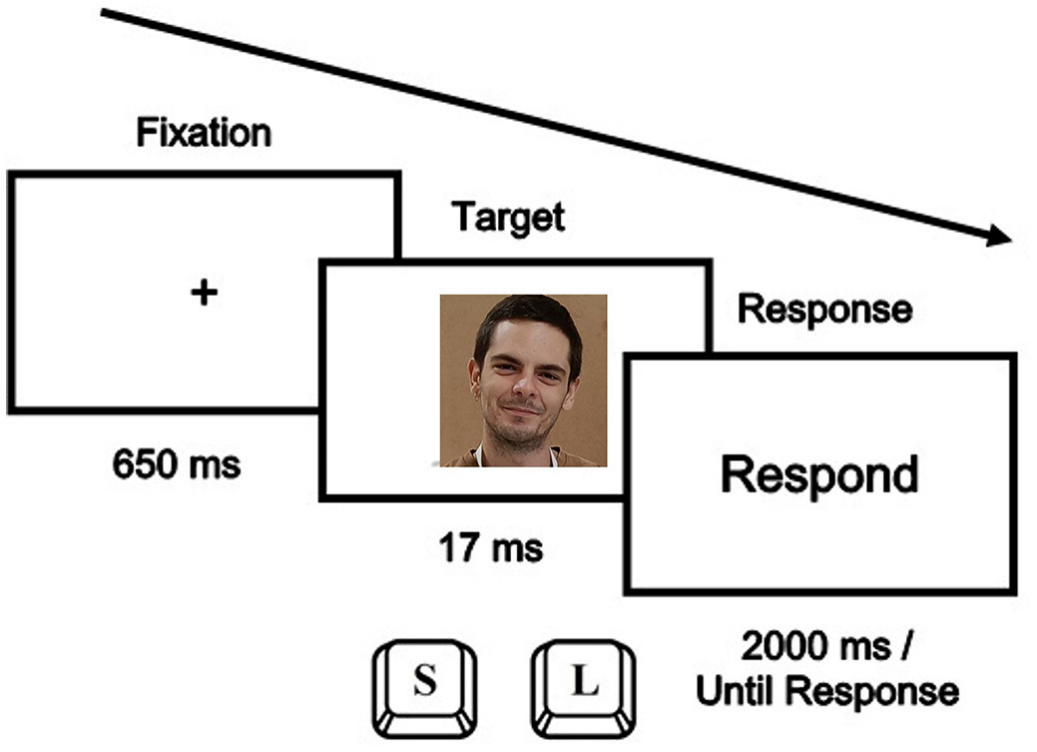
Temporal sequence of events in the gender-face identification task (the face shown is of one of the authors, AC, who gives his permission to the image to be published).

Each block started with six practice trials, in which only male and female faces appeared, and participants received feedback on the accuracy of their responses. Then, an experimental block of 90 trials began with the same 30 faces (10 male, 10 female, and 10 neutral) appearing three times each throughout the block. Figure 1 illustrates the procedure of a single trial. In each experimental trial, a central fixation cross was presented for a randomly varied duration of 650 ms to 1000 ms, which was replaced by the target stimulus for 17 ms. A new display containing the word “respond” appeared in the center of the screen for 2000 ms, or until the participant's response.

##### Name classification by gender task

The design and procedure for the name classification task were very similar to those for face identification, except that now the stimuli were names, and the target presentation time was increased to 33 ms. Likewise, the distribution of trials across the two experimental blocks was identical to the previous task. Both face and name classification tasks comprised Experiment 1, with the order in which they were performed being counterbalanced across participants.

#### Experiment 2 (object-gender classification task and stroop task)

##### Object-gender classification task

This task was included in Experiment 2, and participants were asked to classify objects based on whether they were perceived as stereotypically feminine or masculine. A procedure similar to that used in the name classification task was followed, in terms of the temporal sequence of events, distribution of trials and arrangement of response keys.

##### Stroop task

**Figure 2:**
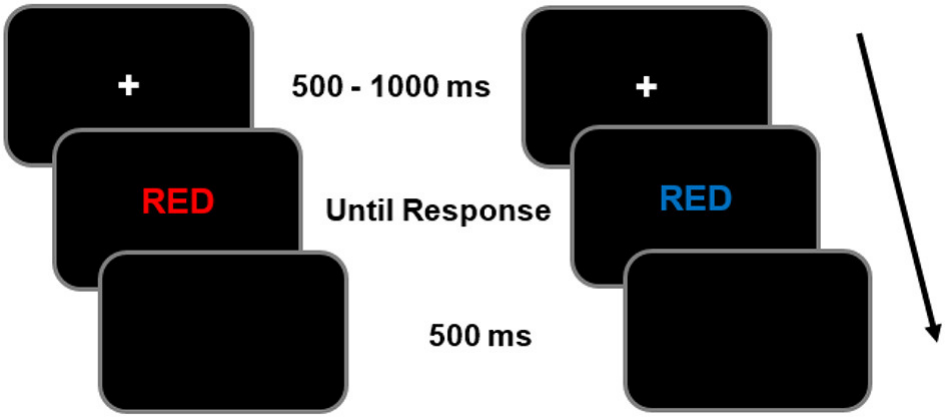
Temporal sequence of events of a congruent trial **(left)** and incongruent **(right)** in the Stroop task.

A computerized version of the Stroop task was used in Experiments 2 and 3. Figure 2 illustrates an example of a trial that began with a central cross as a fixation point with a variable duration of 500–1000 ms. Then, a color word (red, green, blue or yellow) appeared in the center of the screen, and participants had to identify the color of the font with which it was written. For example, if the word “RED” appeared written in a blue font, the correct response would be “blue”. A video game controller with four buttons, each associated with one of the colors, was used to respond. Firstly, participants performed a block of 8 trials to familiarize themselves with the response keys associated with each color. If the error rate of this block exceeded 30%, then these trials were repeated. Otherwise, the task continued with 16 practice trials followed by the experimental block of 48 trials, of which 70% were congruent (i.e., the meaning and color of the word matched, e.g., RED written in red font), while the remaining 30% were incongruent, as both meaning and font color did not match (e.g., RED written in blue font).

#### Experiment 3 (arrow task and stroop task)

##### Arrow task

Participants were asked to respond to the direction of a target arrow, which pointed to the left in 50% of the trials and to the right in the remaining 50%. They had to press the “S” key with their left hand when the arrow pointed to the left, and the “L” key with their right hand when it pointed to the right. The target was preceded by the image of an object that acted as a prime stimulus. The set of stimuli used as primes was the same as that used in the task of classifying objects by gender.

**Figure 3:**
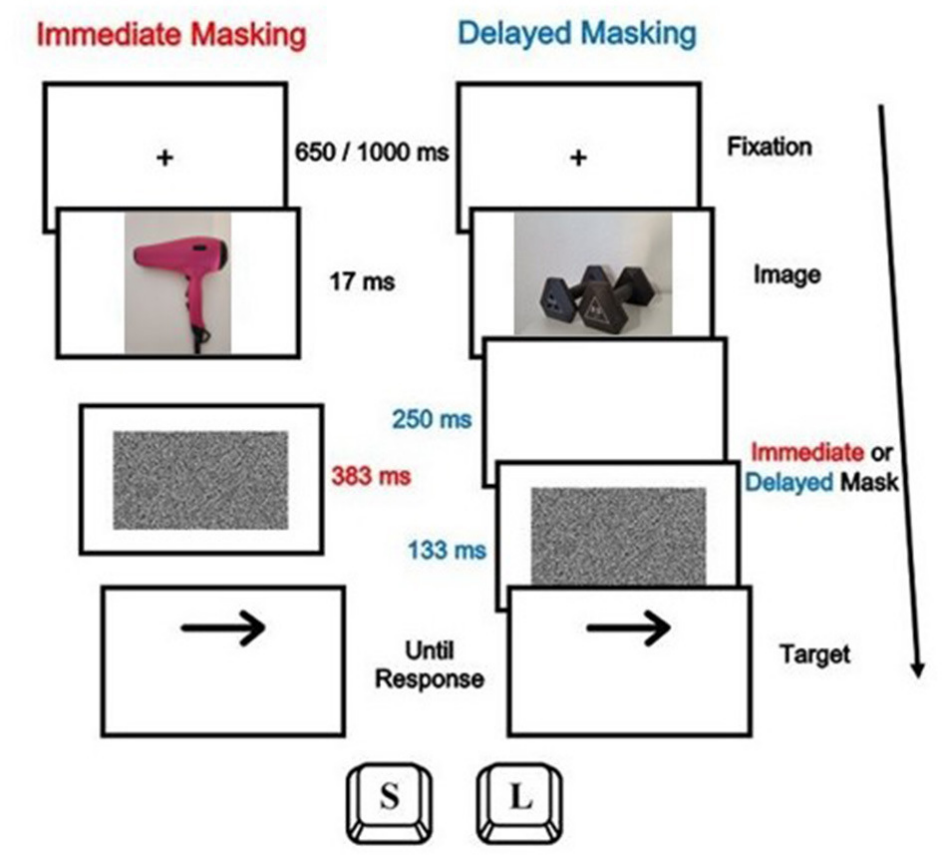
Temporal sequence of events of a trial for both masking conditions in the Arrow Task. On the left incongruent gender-space condition (female- right direction of the arrow), and, on the right, congruent gender-space condition (male-right direction of the arrow).

As can be seen in Figure 3, each trial began with a variable fixation point of 650 or 1000 ms, followed by the image of an object for 17 ms. Participants were not given explicit instructions on how to process the prime stimulus, only to focus on responding to the direction of the arrow. The prime stimuli were either masked for 383 ms (immediate masking condition in 50% of the trials) or were followed by a delay of 250 ms and a mask for 133 ms (delayed masking condition in the remaining 50%). In the first masking condition the immediate presence of a mask prevents them from being consciously perceived, while in the second condition they are clearly visible. Two blocks of 120 trials were administered. In half of each block (60 trials) the arrow was above the fixation, and in the other half below. Each object was presented once in each masking condition. The order of the blocks was counterbalanced, and the trials were randomly displayed. Finally, a recognition test was administered to measure the objective threshold of visibility of the objects preceding the target arrow presentation. Participants were instructed to identify the objects as “feminine” or “masculine” by pressing the B key or the N key, respectively. There was a total of 10 trials for each masking condition.

### Statistical analysis

The Kolmogorov-Smirnov test and Levene's test were conducted to assess normality of data and homogeneity of variance, respectively. The results showed a normal distribution of data and homogeneity of variance in all variables. Latencies larger than 2.5 standard deviations above the means were excluded from the analyses. An alpha of 0.05 was used for tests of statistical significance.

Reaction times (RTs) of correct responses were analyzed in Experiments 1 and 2 as a function of 4 factors in a repeated measures ANOVA: Stimulus Type (names, faces) × Gender-Space Line (congruent block [Female-Male] and incongruent block [Male-Female] × Stimulus Gender (male, female, neutral), as the within-subject factors; and the Gender of participants (male and female) as the between subject factor. The effect of the Gender-Space Line was based on response hand and gender association, such that the left-female/right-male (female-male) will be considered as a congruent schema, and the left-male/right-female (male-female) association as incongruent.

Regarding Experiment 2, an analysis of covariance (ANCOVA) was also conducted, treating Stroop interference (calculated as the difference between the mean RT of incongruent trials and the mean RT of congruent trials) as a continuous covariate variable.

An ANOVA was performed in Experiment 3 with two within-subject factors: Mask (immediate, delayed) × Arrow direction (congruent with Left-Female object/Right-Male object], incongruent with Left-Male object/Right-Female object, and Gender of participants (male and female) as the between subject factor. Preliminary analyses showed that the location of the arrow (above or below the fixation point) did not affect the results, which is why this variable was excluded from the analyses. The Stroop interference results were employed in a linear regression analysis.

Follow-up paired *t*-test comparisons were performed to examine the significant interactions.

The discriminability index was calculated using data from the recognition task in Experiment 3 for the two masking conditions, according to the equation d' = ZHits – ZFA (Macmillan and Creelman, 1991; Russo et al., 2017). Chance-level discrimination when d' = 0.

## Results and discussion

### Experiment 1 (faces and names gender classification tasks)

**Figure 4:**
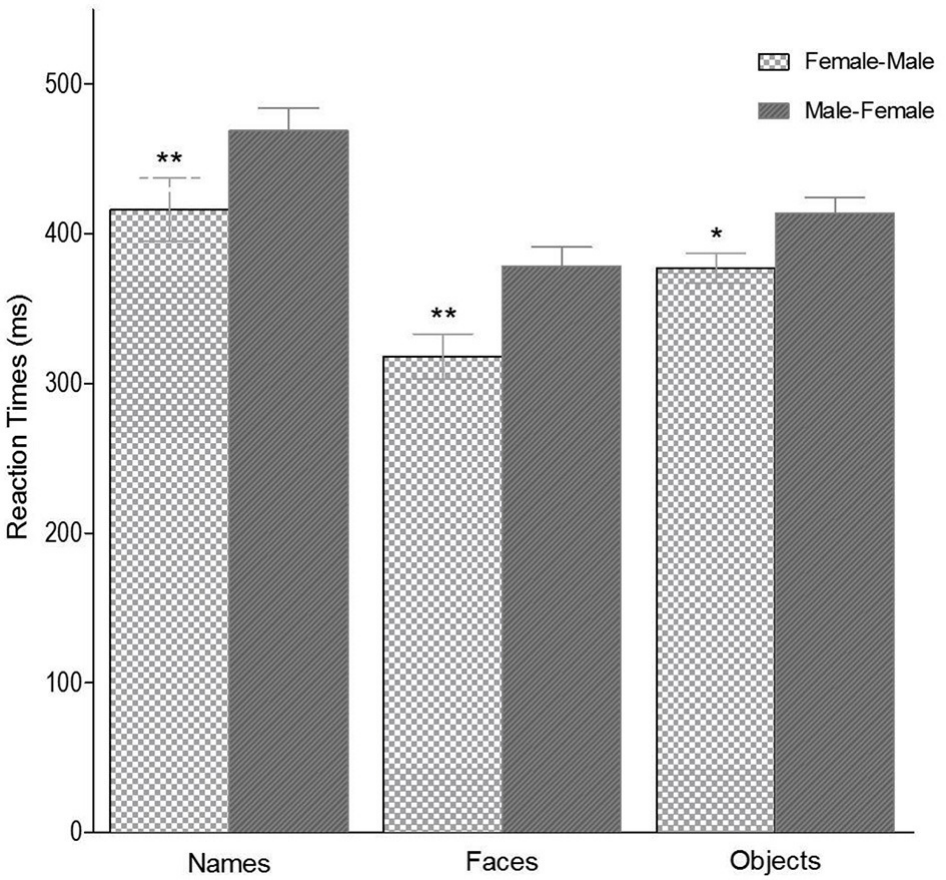
Gender-Space Line effects, Congruent (Female-Male) and Incongruent trials (Male-Female), in the names, faces and objects gender identification task. Error bars represent the standard error of the mean. Simple asterisk means *p* < 0.01. Double asterisk means *p* < 0.001.

The ANOVA showed a main effect of Stimulus Type [*F*_(1, 42)_ = 84.20, *p* < 0.001, η^2^ = 0.671], with shorter response times for faces than for names, 359.40 ms (*SD* = 15.39) and 491.75 ms (*SD* = 12.36), respectively. Also, a main effect of Stimulus Gender [*F*_(2, 42)_ = 101.21, *p* < 0.001, η^2^ = 0.770] was found, with longer response times to classify neutral stimuli (*Mean* = 505.52 ms*, SD* = *15.75*) than for male (*Mean* = 383.67 ms, *SD* = 10.60) and female (*Mean* = 387.26 ms*, SD* = 11.25). The paired comparison *t*-test confirmed the significant differences between the RTs for neutral and male stimuli [*t*_(43)_ = 7.2, *p* = 0.0007] and between neutral and female stimuli [*t*(43) = 6.2, *p* = 0.0008]. No significant differences were observed between male and female stimuli (*p* > 0.05). In addition, there was a significant effect for the Gender-Space Line [*F*_(1, 42)_ = 27.66, *p* < 0.001, η^2^ = 0.400]. Participants showed shorter response times with the left hand to the female and with the right hand to the male stimuli (*Mean* = 404, 80 ms*, SD* = 11.92) than the opposite pattern, left hand-male/right hand-female (*Mean* = 446,17 ms*, SD* = 13.22). These data are graphically represented in Figure 4. No further main effects or interactions were found (*ps* > 0.1).

### Experiment 2 (object gender classification task)

The ANOVA showed a main effect for the Gender-Space Line [*F*_(1, 28)_ = 7.940; *p* = 0.009; η^2^ = 0.221] due to lower response times with the left hand to female objects and with the right hand to male objects (*Mean* = 377.5 ms, *SD* = 14.6), compared to the male-left and female-right association (*Mean* = 404 ms, *SD* = 16.4). This finding is shown in Figure 4. A main effect for Object Gender also emerged [*F*_(2, 56)_ = 72.140, *p* < 0.001, η^2^ = 0.720]. A paired samples *t*-test showed significant differences between the mean response times of the three levels: Female and Male [*t*_(29)_ = −2.66, *p* = 0.012]; Female and Neutral [*t*_(29)_ = −2.66, *p* = 0.012]; Female and Neutral [*t*_(29)_ = −9.99, *p* = 0.012], and Male and Neutral [*t*_(29)_ = −8.36, *p* < 0.01]. Specifically, shorter response times were obtained for Female objects (*Mean* = 352 ms, *SD* = 84.6), than for Male objects (*Mean* = 369.3 ms, *SD* = 84.6), while the longest latency was found for Neutral objects (*Mean* = 447.2 ms, *SD* = 93.6). No other main effects nor interactions were found (*p*s > 0.1), with F_(2, 27)_ = 2.79; *p* = 0.079, η^2^ = 0.172 in the interaction Object Gender x Participant Gender. Regarding Stroop task performance, ANCOVA analysis showed no modulating effect of Inhibition Capacity on the Gender-Space Line effect (*p* > 0.1) in the object classification task. No significant correlations were found (*ps* > 0.1).

The results of Experiments 1 and 2 showed, firstly, that there was a spatial mental representation of faces, names and objects based on their gender. This is consistent with previous research in which a similar representation based on gender was also found (Maass et al., 2009; Zhang et al., 2014; Zarzeczna et al., 2020). Secondly, no relationship was found between this Gender-Space Line effect, measured with a task where the gender of the stimulus was explicitly asked, and the inhibition of distracting information measured by using a Stroop task. These data are in line with those obtained by several investigations (e.g., Hoffmann et al., 2014; Georges et al., 2018), to the extent that the results of the Stroop task did not explain the strength of the SNARC effect, when explicitly asked for the magnitude of the number. Based on these results, a new experiment was conducted to examine the relationship between cognitive inhibition and spatial mental representation of gender, but in this case, participants were instructed to respond to the direction an arrow pointed and not to gender.

### Experiment 3 (arrow task)

**Figure 5:**
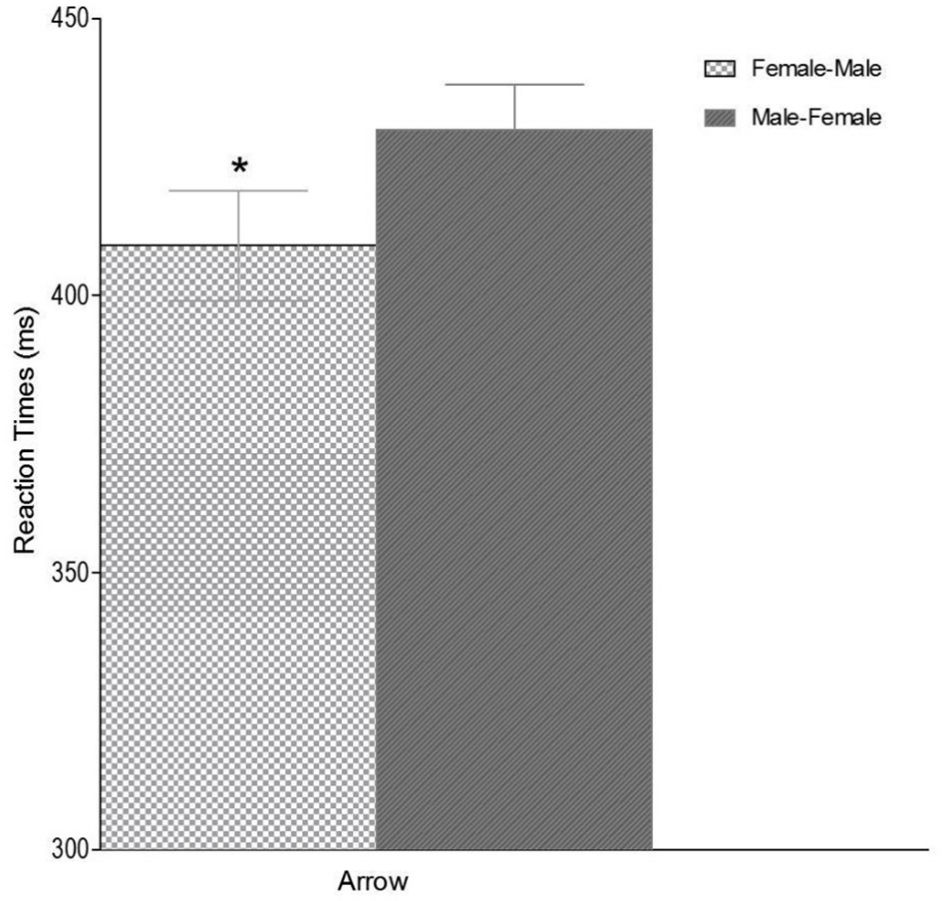
Gender-Space Line effect, Congruent (Female-Male) and Incongruent trials (Male-Female), in Arrow task. Error bars represent the standard error of the mean. Simple asterisk means *p* < 0.05.

The analysis of RTs showed, again, a significant main effect for arrow-direction [*F*_(1, 18)_ = 6.80, *p* = 0.018, η^2^ = 0.270]. As shown in Figure 5, the mean RTs were shorter for the female object/left direction and male/right direction associations or congruent trials (*Mean* = 419.94 ms, *SD* = 10.45), than for the male/left direction and female/right associations or incongruent trials (*Mean* = 428.11 ms, *SD* = 10.37).

The two way interaction Arrow Direction x Mask was not statistically significant [*F*_(1, 18)_ = 0.090, *p* = 0.77, η^2^ = 0.005]. The Arrow Direction effect was significant in the Delayed masking [*t*_(19)_ =2.0, *p* = 0.05] and in the Immediate masking condition [*t*_(19)_ = 2.1, *p* = 0.05].

No other main effects nor interactions were found (*p*s>0.1).

Further correlation analysis showed a high rate of positive correlation between the difference in the average latency in the congruent and incongruent conditions of the Stroop task (the Stroop interference effect), and the average response times in the incongruent male/left direction and female/right direction of arrow conditions, in both delayed and immediate masking conditions, Immediate [*r*_(20)_ = 0.457, *p* = 0.025, critical *r* value = 0.441], and Delayed [*r*_(20)_ = 0.403, *p* = 0.039, critical *r* value = 0.398]. By contrast, the correlation analyses did not show any significant relationship between the Stroop interference and the average response times in the congruent female/left and male/right direction, neither in the Delayed [*r*_(20)_ = 0.31, *p* = 0.178] nor the Immediate masking conditions [*r*_(20)_ = 0.31, *p* = 0.188].

**Figure 6:**
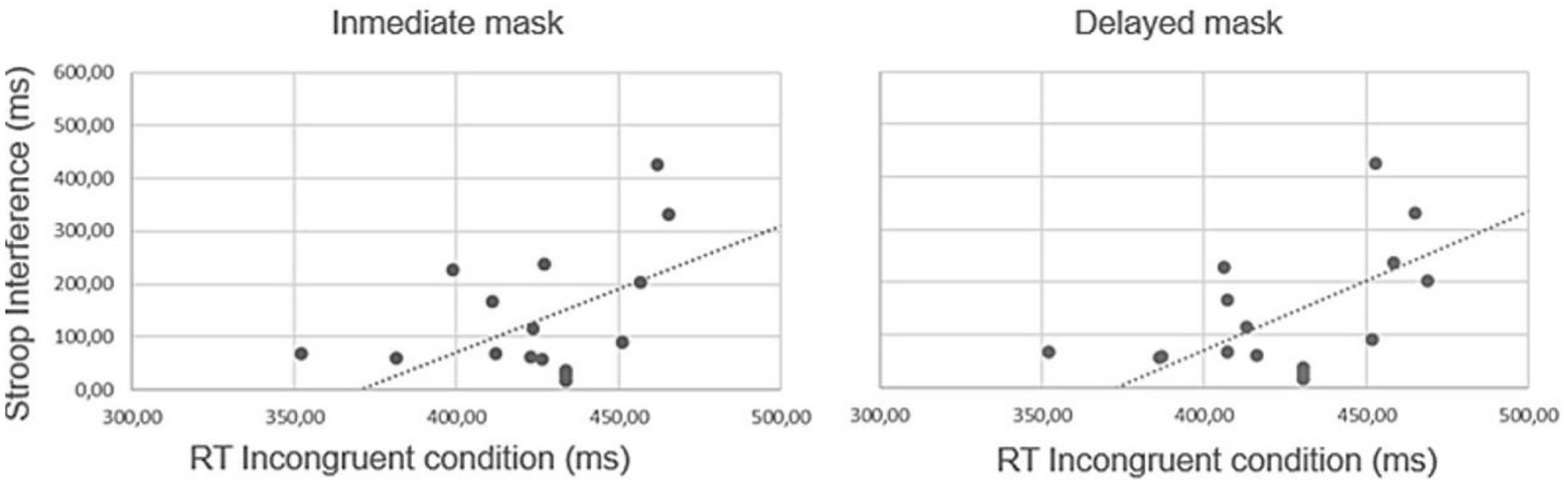
Correlations between Stroop Interference (in ms) and the reaction times (RT) in Incongruent condition (male-left direction of arrow, female-right direction of arrow) as a function of making condition (Immediate and Delayed).

This pattern is graphically represented in Figure 6. Moreover, a linear regression analysis revealed that the Inhibition Capacity was a predictor of response times in incongruent trials, in the immediate masking condition [*R*^2^ = 0.209, *F*_(1, 18)_ = 4.75, *p* = 0.043]. Although it was not found in the delayed masking condition [*R*^2^ = 0.13, *F*_(1, 18)_ = 3.85, *p* = 0.078].

#### Recognition task

The analyses showed a high discriminability index in the delayed masking condition (d' = 0.74), which was significantly higher than chance level [*t* (20) = 7.2, *p* < 0.001], and a low index around chance level [*t* (20) = 0.81, *p* > 0.05] in the immediate masking condition (d' = 0.01).

The results of Experiment 3 again confirmed the occurrence of the spatial representation effect of gender, even when the gender of the stimulus was not task relevant. Moreover, this effect also emerged in the immediate mask condition, when the participant was not aware of the stimulus. These results would indicate that the activation of the spatial mental schema of gender takes place automatically, as would occur in the SNARC effect.

Moreover, coinciding with the findings of Hoffmann et al. (2014) and Georges et al. (2018) in relation to the magnitude of the number and the SNARC effect, a relationship was found between inhibition capacity and the appearance of this effect when the task did not explicitly ask for the identification of the gender of the stimulus.

## General discussion

Based on the SNARC effect and on subsequent studies on the spatial representation of different stimuli, including more social aspects of cognition (Maass et al., 2009; Zhang et al., 2014; Zarzeczna et al., 2020), we raised the possibility that there was also a spatial mental representation of gender. Indeed, in Experiments 1, 2 and 3, a gender-space association effect was observed, in terms of faster responses to the left-female/right-male than the left-male/right-female order. This gender-space association effect was obtained for all three types of stereotyped stimuli (faces, names and objects), extending the effect beyond the mental number line, and the spatial representation of non-numerical stimuli (Santiago et al., 2007; Fuhrman and Boroditsky, 2010; Sellaro et al., 2015). In addition, the spatial representation of gender was clearly biased by writing direction in Western cultures, replicating the same pattern obtained in other studies (Maass et al., 2009; Shaki et al., 2009; Suitner and Maass, 2016).

Although Maass and colleagues had already explored the spatial mental representation of gender (SAB effect), it should be noted that they examined this effect by means of action scenes, which involve a subject-object order. They observed an opposite pattern to the one found in the present study, with males on the left and females on the right (Maass et al., 2009). It is not, therefore, a matter of mentally representing a single object in space, but a scene that includes actions that require the active participation of a subject. Thus, it is possible that the mental schema activated for action scenes differs from that activated when single stimuli are presented.

The fact that the SNARC effect was not observed in all participants by Maass et al. (2009) led some authors to consider whether individual differences in aspects, such as working memory capacity, age or the ability to inhibit irrelevant information, could somehow influence the strength of number-space associations. Hoffmann et al. (2014) reported that age, Stroop interference and processing speed conditioned the magnitude of the SNARC effect. The role played by inhibition capacity in the inter-individual variability was later confirmed by Georges et al. (2018), who also emphasized the importance of the nature (explicit vs. implicit) of the main task.

To the extent that these issues have yet to be addressed in the context of gender-space association representation, the present study aims to explore whether the gender-space line effect could also be modulated by the inhibitory ability of participants, and/or by the nature of the gender task. The data from the third experiment showed that the gender-space line also emerges when identifying the gender of an object was not relevant to the task (but rather indicating the direction of an arrow). In this case, variability in cognitive inhibition, as measured through the Stroop task, would explain part of the appearance of the effect, but not when participants were instructed to classify an object as masculine or feminine (as in Experiment 2). In other terms, the (greater or lesser) capacity for inhibition did not influence the strength of occurrence of the gender-space line effect when the gender task was of an explicit nature, defined in terms of responding by attending to gender. However, in the arrow task, the presentation of an object before the target arrow appeared would automatically induce the activation of the female-male schema, even though one did not respond to the gender, but to the arrow. In incongruent trials, the response hand based on this mental schema would interfere with the target response hand (e.g., female object-left hand/rightward arrow-right hand). Presumably, participants with a greater ability to inhibit the gender-space line (and associated responses) will be more efficient to respond to the actual demands of the task, compared to those with lower cognitive inhibition ability (or efficiency), who showed a stronger gender-space association effect. This response pattern would be in line with the above-mentioned works (see also Hoffmann et al., 2014; Georges et al., 2018; Xiang et al., 2022).

It is important to note that the results showed in the gender stereotypes questionnaire (Castillo and Montes, 2007) suggest positive valence of the female gender in our sample. Hence, the pattern positive valence-right, negative valence-left seems to be weaker than the female-left, male-right pattern showed in our study. The present study also provides evidence on the activation of a spatial mental representation for gender under subliminal perceptual conditions, that is, when the participant is not aware of the prime object (immediate masking condition). This fact reinforces the idea of the automatic nature of this process.

Although all the similarities and differences between the spatial mental representation of number and gender remain unclear, it must be recognized that the variable used in this study (gender) cannot be measured objectively, as is the magnitude of a number. This fact limits the comparison of studies, both in the design and in the theoretical conclusions of the experiments. The use of stimuli in which gender can be conceived in a more objective and uniform way (e.g., grammatical gender of Spanish words) would fix the limitations and help to compare both effects. The task of classifying words according to grammatical gender will also allow us to explore the extent to which the spatial association of gender is related to subjective and cultural aspects. By manipulating the relationship between word meaning and gender stereotypes and roles, we could examine, for example, whether it takes longer to respond with the right hand to a grammatically masculine word but belonging to the feminine gender, than to a grammatically masculine word and belonging to the masculine gender. The results of this experiment would help to better understand the effect found in the Object Classification Task, in which participants identify feminine (vs. masculine) objects significantly faster, an effect that was not observed when classifying names and faces.

Further research is needed to explain the cognitive processes that underlie the gender-space line effect. It would be interesting to observe through neuroimaging techniques the cortical areas involved, both in a face, name and object gender classification task. This would not only provide information about which cortical areas are activated during the occurrence of the effect but would also provide clues about the cognitive mechanisms behind the gender-spatial association and its strength of occurrence. In addition, it will be necessary to explore the implicit self-categorization of gender to identify any possible relationships between the gender-space association and the feeling of group belonging (ingroup vs. outgroup) in line with what happens in the SOSC effect.

Finally, in future research these results could be extended to include different stimuli to determine, for instance: what would occur if what was classified were the faces of both young and old men and women; whether this would have any relationship with agentic traits as occurred with Maass et al. (2009); and, whether there would be a relationship between the spatial mental representation of gender and time. Trying to answer these questions would provide us with more information about the basis of stereotypes, their relationship with prejudices, with implicit associations that we are not aware of and that may be modulating our patterns or actions. By designing activities similar to the tasks described in this study, values, negative stereotypes or prejudices could probably be addressed in early childhood, and/or in people with antisocial behavior.

## Data availability statement

The raw data supporting the conclusions of this article will be made available by the authors, without undue reservation.

## Ethics statement

The studies involving human participants were reviewed and approved by the University of Almería Human Research Ethics Committee and conducted in accordance with the Declaration of Helsinki. The patients/participants provided their written informed consent to participate in this study.

## Author contributions

AC, IC, and CN developed the concept and the design of the experimental work and were responsible for writing the manuscript. AC, IC, DÁ, SF, and CN participated in the implementation of the experimental tasks, data collection, and data analyses. All authors supervised the processes of accomplishing the study, substantially contributed to the interpretation of data, to writing and reviewing the manuscript, as well as to approving the final version of the manuscript.
