# Supplementary material for: Cognitive inhibition abilities explain inter-individual variability in gender-space associations
Source: Front Psychol. 2023 May 17;14:1130105. doi: 10.3389/fpsyg.2023.1130105 (PMC10229869; doi:10.3389/fpsyg.2023.1130105)
Supplement: Supplementary file 1 [file Data_Sheet_1.pdf]

Images used in experiments 2 and 3.

<u>Femenine objects</u>

Image of a bouquet

Result in the form: 2,77 (SD = 0,94)
(1= most feminine; 4= neutral; 7= most masculine)
Valence: Positive
Social power: 5 (SD = 1,78)
(1= no social power; 5= maximum social power)
Grammatical gender: Feminine

Image of a pincushion

Result in the form: 2,22 (SD = 0,91)
(1= most feminine; 4= neutral 10= most masculine)
Valence: Positive
Social power: 2 (SD = 1,64)
(1= no social power; 5= maximum social power)
Grammatical gender: Masculine

Image of a ring

Result in the form: 2,14 (SD = 1)
(1= most feminine; 4= neutral; 7= most masculine)
Valence: Positive
Social power: 5 (SD = 0,54)
(1= no social power; 5= maximum social power)
Grammatical gender: Masculine

Image of a lipstick

Result in the form: 1,74 (SD = 0,9)
(1= most feminine; 4= neutral; 7= most masculine)
Valence: Positive
Social power: 3 (SD = 1)
(1= no social power; 5= maximum social power)
Grammatical gender: Masculine

Image of a teapot

Result in the form: 2,65 (SD = 0,9)
(1= most feminine; 4= neutral; 7= most masculine)
Valence: Positive
Social power: 3 (SD = 1,14)
(1= no social power; 5= maximum social power)
Grammatical gender: Feminine

Image of a heel

Result in the form: 1,51 (SD = 0,7)
(1= most feminine; 4= neutral; 7= most masculine)
Valence: Positive
Social power: 3 (SD = 0,83)
(1= no social power; 5= maximum social power)
Grammatical gender: Masculine

Image of a fan

Result in the form: 2,28 (0,98)
(1= most feminine; 4= neutral; 7= most masculine)
Valence: Positive
Social power: 3 (SD = 1,09)
(1= no social power; 5= maximum social power)
Grammatical gender: Masculine

Image of a heel

Result in the form: 1,51 (SD = 0,56)
(1= most feminine; 4= neutral; 7= most masculine)
Valence: Positive
Social power: 3 (SD = 1)
(1= no social power; 5= maximum social power)
Grammatical gender: Masculine

Image of a nail polish

Result in the form: 2,31 (SD = 1)
(1= most feminine; 4= neutral; 7= most masculine)
Valence: Positive
Social power: 3 (SD = 1,34)
(1= no social power; 5= maximum social power)
Grammatical gender: Masculine

Masculine objects

Image of a helmet

Result in the form: 5,45 (SD = 1,06)
(1= most feminine; 4= neutral; 7= most masculine)
Valence: Positive
Social power: 2 (SD = 1,14)
(1= no social power; 5= maximum social power)
Grammatical gender: Masculine

Image of a gun

Result in the form: 5,57 (SD = 1,2)
(1= most feminine; 4= neutral; 7= most masculine)
Valence: Negative
Social power: 5 (SD = 0,54)
(1= no social power; 5= maximum social power)
Grammatical gender: Feminine

Image of a truck

Result in the form: 6 (SD = 0,97)
(1= most feminine; 4= neutral; 7= most masculine)
Valence: Neutral
Social power: 2 (SD = 0,83)
(1= no social power; 5= maximum social power)
Grammatical gender: Masculine

Image of a axe

Result in the form: 5,4 (SD = 1)
(1= most feminine; 4= neutral; 7= most masculine)
Valence: Positive
Social power: 3 (SD = 1,14)
(1= no social power; 5= maximum social power)
Grammatical gender: Feminine

Result in the form: 5,77 (SD = 1,08)
(1= most feminine; 4= neutral; 7= most masculine)
Valence: Negative
Social power: 2 (SD = 0,89)
(1= no social power; 5= maximum social power)
Grammatical gender: Feminine

Image of a wrench

Image of a electric screwdriver

Result in the form: 5,34 (SD = 1,18)
(1= most feminine; 4= neutral; 7= most masculine)
Valence: Positive
Social power: 3 (SD = 0,7)
(1= no social power; 5= maximum social power)
Grammatical gender: Masculine

Image of weights

Result in the form: 5,34 (SD = 0,96)
(1= most feminine; 4= neutral; 7= most masculine)
Valence: Positive
Social power: 3 (SD = 1,09)
(1= no social power; 5= maximum social power)
Grammatical gender: Feminine

Image of a wrench

Result in the form: 5,48 (SD = 0,86)
(1= most feminine; 4= neutral; 7= most masculine)
Valence: Positive
Social power: 3 (SD = 1,09)
(1= no social power; 5= maximum social power)
Grammatical gender: Feminine

Image of a shaving razor

Result in the form: 5,85 (SD = 1,06)
(1= most feminine; 4= neutral; 7= most masculine)
Valence: Neutral
Social power: 1 (SD = 1,87)
(1= no social power; 5= maximum social power)
Grammatical gender: Feminine

Image of a boot

Result in the form: 5,48 (SD = 0,91)
(1= most feminine; 4= neutral; 7= most masculine)
Valence: Positive
Social power: 1 (SD = 1,87)
(1= no social power; 5= maximum social power)
Grammatical gender: Masculine

Objects not related to any gender

Image of a padlock

Result in the form: 4,02 (SD = 0,38)
(1= most feminine; 4= neutral; 7= most masculine)
Valence: Neutral
Social power: 1 (SD = 1)
(1= no social power; 5= maximum social power)
Grammatical gender: Masculine

Image of a wrench

Result in the form: 3,97 (SD = 0,16)
(1= most feminine; 4= neutral; 7= most masculine)
Valence: Positive
Social power: 1 (SD = 1,51)
(1= no social power; 5= maximum social power)
Grammatical gender: Masculine

Image of a CD

Result in the form: 4 (SD = 0,24)
(1= most feminine; 4= neutral; 7= most masculine)
Valence: Positive
Social power: 1 (SD = 1,3)
(1= no social power; 5= maximum social power)
Grammatical gender: Masculine

Image of a scissors

Result in the form: 4 (SD = 0)
(1= most feminine; 4= neutral; 7= most masculine)
Valence: Neutral
Social power: 1 (SD = 0,89)
(1= no social power; 5= maximum social power)
Grammatical gender: Feminine

Image of a mug

Result in the form: 4 (SD = 0,34)
(1= most feminine; 4= neutral; 7= most masculine)
Valence: Neutral
Social power: 3 (SD = 1,09)
(1= no social power; 5= maximum social power)
Grammatical gender: Feminine

Image of coins

Result in the form: 3,94 (SD = 0,41)
(1= most feminine; 4= neutral; 7= most masculine)
Valence: Positive
Social power: 4 (SD = 0,83)
(1= no social power; 5= maximum social power)
Grammatical gender: Feminine

Image of a microphone

Result in the form: 4 (SD = 0,64)
(1= most feminine; 4= neutral; 7= most masculine)
Valence: Positive
Social power: 3 (SD = 0,83)
(1= no social power; 5= maximum social power)
Grammatical gender: Masculine

Image of a magnifying

Result in the form: 4,05 (SD = 0,33)
(1= most feminine; 4= neutral; 7= most masculine)
Valence: Positive
Social power: 1 (SD = 1)
(1= no social power; 5= maximum social power)
Grammatical gender: Feminine

Image of a clip

Result in the form: 3,94 (SD = 0,33)
(1= most feminine; 4= neutral; 7= most masculine)
Valence: Neutral
Social power: 1 (SD = 0,89)
(1= no social power; 5= maximum social power)
Grammatical gender: Masculine

Note: The images used are not shown in this supplementary material in order to protect the copyright.
