# Supplementary material for: Cognitive inhibition abilities explain inter-individual variability in gender-space associations
Source: Front Psychol. 2023 May 17;14:1130105. doi: 10.3389/fpsyg.2023.1130105 (PMC10229869; doi:10.3389/fpsyg.2023.1130105)
Supplement: Supplementary file 2 [file Data_Sheet_2.PDF]

Name	Form score	Gender
Laura	0'8	Female
Nuria	0'9	Female
Eva	1,0	Female
Carolina	1,0	Female
Marta	0'9	Female
Sofía	0'8	Female
Lola	0'9	Female
Rocío	0'9	Female
Isabel	0'8	Female
Ángela	0'9	Female
Azul	4'6	Unisex
Gael	6'6	Unisex
Santana	5'3	Unisex
Paris	4,0	Unisex
Cruz	4'3	Unisex
Pau	5'6	Unisex
Milán	5'6	Unisex
Luján	4'5	Unisex
Dani	6'65	Unisex
Ariel	3'3	Unisex
Santiago	9,0	Male
Javier	8'9	Male
Rubén	9,0	Male
Gonzalo	9'1	Male
Carlos	8'9	Male
Pedro	9'2	Male
Mateo	8'9	Male
Diego	9,0	Male
Hugo	9,0	Male
Pablo	10,0	Male

0 = Most Femenine
10 = Most Masculine
